# Socioeconomic inequality in oral health behavior in Iranian children and adolescents by the Oaxaca-Blinder decomposition method: the CASPIAN- IV study

**DOI:** 10.1186/s12939-016-0423-8

**Published:** 2016-09-14

**Authors:** Saeid Safiri, Roya Kelishadi, Ramin Heshmat, Ali Rahimi, Shirin Djalalinia, Anoosheh Ghasemian, Ali Sheidaei, Mohammad Esmaeil Motlagh, Gelayol Ardalan, Morteza Mansourian, Hamid Asayesh, Mahdi Sepidarkish, Mostafa Qorbani

**Affiliations:** 1 Managerial Epidemiology Research Center, Department of Public Health, School of Nursing and Midwifery, Maragheh University of Medical Sciences, Maragheh, Iran; 2 Road Traffic Injury Research Center, Department of Statistics & Epidemiology, Tabriz University of Medical Sciences, Tabriz, Iran; 3 Child Department of Pediatrics, Child Growth and Development Research Center, Research Institute for Primordial Prevention of Non-communicable Disease, Isfahan University of Medical Sciences, Isfahan, Iran; 4 Chronic Diseases Research Center, Endocrinology and Metabolism Population Sciences Institute, Tehran University of Medical Sciences, Tehran, Iran; 5 School of Humanities and Tourism Management, Bangkok University, Bangkok, Thailand; 6 Development of Research & Technology Center, Deputy of Research and Technology, Ministry of Health and Medical Education, Tehran, Iran; 7 Dental School, Tehran University of Medical Sciences, Tehran, Iran; 8 Department of Epidemiology and Biostatistics, Shahid Beheshti University of Medical Science, Tehran, Iran; 9 Department of Pediatrics, Ahvaz Jondishapour University of Medical Sciences, Ahvaz, Iran; 10 Department of Health Education, School of Public Health, Iran University of Medical Sciences, Tehran, Iran; 11 Department of Medical Emergencies, Qom University of Medical Sciences, Qom, Iran; 12 Department of Epidemiology and Reproductive Health, Reproductive Epidemiology Research Centre, Royan Institute for Reproductive Biomedicine, ACECR, Tehran, Iran; 13 Department of Community Medicine, School of Medicine, Alborz University of Medical Science, Karaj, Iran; 14 Endocrinology and Metabolism Research Center, Endocrinology and Metabolism Clinical Sciences Institute, Tehran University of Medical Sciences, Tehran, Iran

**Keywords:** Adolescents, Children, Iran, Inequality, Oaxaca-Blinder decomposition, Oral health

## Abstract

**Background:**

The present study set to describe the socioeconomic inequality associated with oral hygiene behavior among Iranian pediatric population.

**Methods:**

A representative sample of 13486 school students aged 6–18 years was selected through multistage random cluster sampling method from urban and rural areas of 30 provinces in Iran. Principle Component Analyses (PCA) correlated variables summarized as socioeconomic status (SES). Association of independent variables with tooth brushing was assessed through logistic regression analysis. Decomposition of the gap in tooth brushing between the first and fifth SES quintiles was assessed using the counterfactual decomposition technique. To assess the relation between tooth brushing and each socioeconomic category, Concentration Index (C) and the slope index of inequality (SII) were used, representing the linear regression coefficient.

**Results:**

The participation rate was 90.6 % (50.7 % boys and 75.6 % urban inhabitants). The mean age of participants was 12.47 ± 3.36 years. The frequency of tooth brushing increased across SES quintiles, prevalence of tooth brushing between the first and fifth quintile, under 20 % difference, increased from 58.22 (95 % CI: 56.24,60.20) to 78.61 (95 % CI: 77.00,80.24). Only 3 % of the difference is explained by the factors considered in the study, and 17 % remained unknown. Residence area, family size, and smoking status made a significant contribution to the gap between the first and last SE groups. Residence area [ −2.01 (95 % CI: −3.46, −0.55)] was along the maximum levels of gaps between SE categories.

**Conclusions:**

The findings revealed a socio-economic inequality in oral health behavior in Iranian children and adolescents. Also, factors influencing oral health are addressed to develop and implement complementary public health actions.

## Background

Through widespread health measures, families with lower socioeconomic status (SES) have higher rates of diseases and disabilities such as cardiovascular disease, cancer, diabetes, and birth defects [1–3]. These health inequalities originate from different factors such as health behavior since lower socioeconomic individuals have possibly more unhealthy behaviors [1–3].

According to Surgeon General’s Report on Oral Health [4], there are disparities in oral health, where people with lower SES are more susceptible to oral diseases such as dental caries, periodontal disease, and oral cancer. Studies indicate significant increase in the differences in the oral health status between individuals with high and low SES [5]. Also, children with different SES have various forms of food consumption and oral health practices such as tooth brushing. These differences could be considered as mediators of the relationship between SES and oral health, which are essential to address the oral health inequality and improvement of children [6].

There are limited evidence-based studies on measuring oral health inequalities. Most studies have only evaluated the association between lower SES and caries, without assessing the reason for such associations [7–11]. Therefore, it is very little known about specific oral hygiene behaviors such as tooth brushing in families with different SES [12, 13]. According to a national study, Iran has equal or higher oral health habits compared with other countries specially in tooth brushing; the frequency was found more in girls than boys and more in urban areas than rural areas [14].

There is a strong need for studies to evaluate the relationship between SES and oral health for identifying particular behavioral factors associated with SES, contributing to the risk of dental caries. Thus, the present study set to describe the socioeconomic inequality associated with oral hygiene behavior among children and adolescents populations in Iran. It is believed that the results may help to support effective evidence-based policies and interventions to improve oral health status in Iran.

## Methods

The findings are derived from the results of fourth round of comprehensive national survey of a school-based surveillance system entitled “Childhood and Adolescence Surveillance and Prevention of Adult Non-communicable Disease” (CASPIAN-IV) study (2011–2012). The details of the study has been previously described [15], here, essentials are pointed in brief.

### Study population

To assess the socioeconomic inequality in oral health behavior of Iranian children and adolescents, the data of 13,486 students aged 6–18 years were used, selected through multistage cluster sampling method from rural and urban areas of 30 provinces of Iran.

Eligible schools for the study were stratified according to information bank of Ministry of Education through multistage cluster sampling method (48 clusters of 10 students in each province). Stratification was performed according to school grade (elementary, middle-, and high school) and residence area (urban, rural). Three targeted age groups were; 6–9.9, 10–13.9, and 14–18 years. Considering the potential probability for loss of samples and confound data, the sample reached 14,880. Participants who had complete data were included in the study.

### Data gathering

A trained team of expert health care providers conducted all examinations and inquiry processes under standard protocols and calibrated instruments. Following the World Health Organization (WHO), global school-based student health survey (GHSH) instructions, the data were recorded in checklists, and validated questionnaires were completed for all participants. To assess the highest data quality in multi-center data gathering, all different levels of quality assurance were exactly supervised by Data and Safety Monitoring Board [15].

#### Definition of terms

- **Oral health behaviors**: Oral health behaviors refer to tooth brushing.
- **Demographic information**: Demographic information includes the age, sex, residence area, birth order, family based characteristics, family history of chronic diseases (hypertension, dyslipidemia, diabetes, and obesity), parental level of education, possessing a family private car, and type of home, completed for all participants through an interview with parents or children.
- **Socioeconomic status** (**SES**): To determine the SES of participants, the methodology approved previously in the Progress in the International Reading Literacy Study (PIRLS) for Iran was used. Using principle component analysis (PCA), parents’ education, parents’ job, possessing private car, school type (public/private), type of home (private/rented), and having a personal computer variables were summarized under one main component, categorized into five quintiles. Through an ascending grade, the first quintile was defined as the “lowest SES” and the fifth quintile as the “highest SES” groups.
- **Smoking status**: Smoking was categorized into three groups; active, passive, and smoking exposure. A person who smoked at least one cigarette a day (seven cigarettes per week) was considered an active smoker. Students who reported smoker people in their living environment were considered passive smokers. Smoking exposure was defined as active or passive smoking or both.
- **Tooth brushing**: General characteristics of the participants were categorized under the categories of self-reported frequency of tooth brushing including more than once a day, once a day, once a week, rarely, and never [14, 16]. For statistical analysis, tooth brushing was considered as a binary variable; more than once a day and once a day were considered as positive tooth brushing behaviors, and other options were analyzed as negative tooth brushing behaviors. More details regarding the variables’ scale are given in Appendix.

### Ethical concerns

The study protocol was reviewed and approved by Ethics Committees of Tehran University of Medical Sciences and Isfahan University of Medical Sciences. Participation for the invited was voluntary. After compete explaining the study aims and protocols, written consent and verbal assent were obtained from the students.

### Statistical analysis

To provide practical information for better health planning, to study the differences between the groups, to determine practical wage differences between two groups explained by group differences in productivity characteristics, and also to clear a residual part that cannot be accounted by such differences in wage determinants, Blinder–Oaxaca decomposition was used for linear regression models [17–19]. This method is based on two regression models, fitted separately for the two population groups (in this study, high and low-economic groups) [20].

1 $$ \mathrm{Y}\mathrm{H} = {\upbeta \mathrm{X}}_{\mathrm{H}} + {\upvarepsilon}_{\mathrm{H}} $$

2 $$ \mathrm{YL} = {\upbeta \mathrm{X}}_{\mathrm{L}} + {\upvarepsilon}_{\mathrm{L}} $$

Y is the outcome variable; β is the coefficient including the intercept; X is the explanatory variable, and ε is the error. The gap between the two groups is:

3 $$ {\overline{\mathrm{y}}}_{\mathrm{H}} - {\overline{\mathrm{y}}}_{\mathrm{L}} = \left({\overline{\mathrm{X}}}_{\mathrm{H}}-{\overline{\mathrm{X}}}_{\mathrm{L}}\right){\upbeta}_{\mathrm{H}}+{\overline{\mathrm{X}}}_{\mathrm{L}}\left({\upbeta}_{\mathrm{H}}-{\upbeta}_{\mathrm{L}}\right) $$

and

4 $$ {\overline{\mathrm{y}}}_{\mathrm{L}} - {\overline{\mathrm{y}}}_{\mathrm{H}} = \left({\overline{\mathrm{X}}}_{\mathrm{H}}-{\overline{\mathrm{X}}}_{\mathrm{L}}\right){\upbeta}_{\mathrm{L}}+{\overline{\mathrm{X}}}_{\mathrm{H}}\left({\upbeta}_{\mathrm{H}}-{\upbeta}_{\mathrm{L}}\right) $$

The first part of the right hand side of the above equations is the observable difference in the variables in the two groups (the endowment or explained component), and the second part is related to the differences in the variable coefficients in the two groups (the coefficient or unexplained component). This technique divides the gap between the mean values of an outcome into two components. The “explained or endowment” component arises because of differences in the groups’ characteristics, such as differences in region or family size. An “unexplained or coefficient” component is attributed to different influences of these characteristics in each group [21]. To perform the decomposition, a logistic regression model was constructed with independent variables in each economic group to determine the regression coefficients (β) as the main effect and its interaction with other independent variables.

Using Principle Component Analyses (PCA), variables including parents’ education, parents’ job, possessing private car, school type (public/private), type of home (private/rented), and having personal computer are summarized as SES [22, 23]. Association of independent variables with tooth brushing is assessed through logistic regression analysis and presented by crude and adjusted OR (95 % CI). Decomposition of the gap in tooth brushing between the first and fifth quintiles of SES was evaluated using the counterfactual decomposition technique which is widely used to study mean outcome differences between groups [18, 24, 25].

To investigate the association of tooth brushing in each socioeconomic category, on the basis of the distribution of tooth brushing versus the distribution of SES, Concentration Index (C) was used, showing how SES inequality in some health outcome variables exists and how distributed at one point [26, 27]. The Slope Index of Inequality (SII) represented the linear regression coefficient; it reveals the relation between the levels of frequency of tooth brushing in each socioeconomic category hierarchical ranking. Hence, targeted variable is created from a series of values according to different SES categories along with a range, and all individuals in population changes are considered along with different SES categories [27, 28]. Statistical measures were assessed using survey data analysis methods in the Stata version 11.1 (Stata Corporation, College Station, TX, USA). Using the method described by Jann [29], the Oaxaca command was ran in version 10 of the Stata software (Stata Corporation, College Station, Texas). *P* < 0.05 was considered as statistically significant.

## Results

From 14880 invited students, 13486 participants completed all required data (participation rate: 90.6 %). The average age range was 12.47 ± 3.36 years, without any significant difference between girls and boys. There were 6640 (49.2 %) girls and 75.6 % urban area residents.

**Fig. 1:**
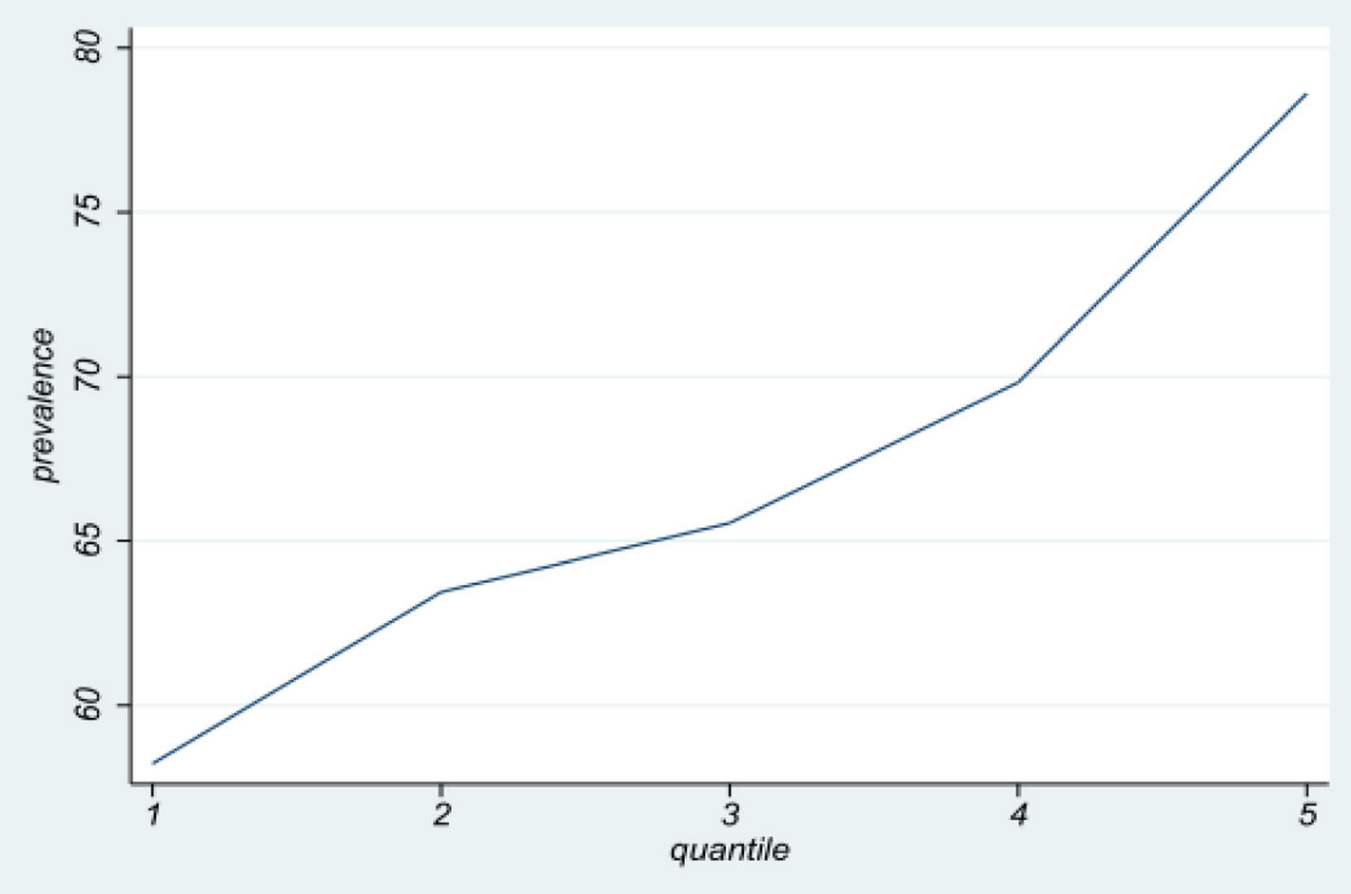
The prevalence of tooth brushing according to socioeconomic quintiles in Iranian children and adolescents: the CASPIAN IV study

The prevalence of tooth brushing had an ascending trend according to socioeconomic quintiles (Fig. 1).

Results revealed that through an increasing pattern of tooth brushing prevalence along with the SES quintiles, tooth brushing prevalence between the first and fifth quintile, under 20 % difference, increased from 58.22 (95 % CI: 56.24, 60.20) to 78.61 (95 % CI: 77.00, 80.24). SII presented an adverse association between SES and oral health-related behaviors (coefficient: −0.24 (95 % CI: −0.34, −0.14)). Moreover, based on the estimated concentration index of 0.06 (SD = 0.003), poor oral health-related behaviors were most probable in low socioeconomic levels. Table 1 shows SES inequality in tooth brushing prevalence in Iranian children and adolescents.

**Table 1:** Socioeconomic inequality in teeth brushing prevalence in Iranian children and adolescents: the CASPIAN IV study

Outcome	Q1 Prevalence [95 % CI]	Q2 Prevalence [95 % CI]	Q3 Prevalence [95 % CI]	Q4 Prevalence [95 % CI]	Q5 Prevalence [95 % CI]	Total Prevalence [95 % CI]	SII [95 % CI]	C (SD)
Tooth brushing	58.22 (56.24,60.20)	63.45 (61.26,65.59)	65.54 (63.43,67.58)	69.83 (67.6,71.97)	78.61 (77.00,80.24)	66.99 (65.72,68.24)	−0.24 (−0.34,−0.14)	0.06 (0.003)

*CI* confidence interval, *Q* quantile, *SII* slope index of inequality, *C* concentration index, *SD* standard deviation

Considering the analysis of independent variables, individuals in quintile 5 families had significantly higher tooth brushing prevalence compared with those counterparts in quintile 1 (OR: 2.71; 95 % CI: 2.39, 3.07). Participants with higher order of birth (third and more), larger family size (>4), compared with their counterparts, had less prevalence of oral health behaviors (24 % and 22 %, respectively).

As Also, living in rural areas was adversely associated with less prevalence of tooth brushing (OR:0.67; 95 % CI: 0.61, 0.72). And the prevalence of tooth brushing was 2.31 (95 % CI: 2.15, 2.49) times more in girls. There was a less chance of increase in tooth brushing with increase of population age (OR: 1.04; 95 % CI: 1.02, 1.05). All categories of smoking status increased the chance of poor oral health behaviors’ (P for trend < 0.001) (Table 2).

**Table 2:** Association of independent variables and teeth brushing in logistic regression analysis

Variables	Crude OR (95 % CI)	*P*-value	Adjusted OR (95 % CI)	*P*-value
SES (Q1)				
Q2	1.28 (1.14, 1.43)	<0.001	1.19 (1.05,1.35)	< 0.001
Q3	1.40 (1.25,1.57)	< 0.001	1.30 (1.14,1.48)	< 0.001
Q4	1.71 (1.52,1.92)	<0.001	1.53 (1.32,1.78)	<0.001
Q5	2.71 (2.39, 3.07)	< 0.001	2.45 (2.08,2.90)	< 0.001
Birth order (first)				
Second	0.90 (0.82,0.98)	0.03	0.97 (0.88,1.08)	0.69
Third	0.74 (0.66,0.82)	<0.001	0.89 (0.78,1.01)	0.09
Fourth and more	0.76 (0.68,0.84)	< 0.001	1.01(0.89,1.15)	0.80
Sex (Boy)				
Girl	2.31(2.15, 2.49)	<0.001	2.40 (2.15,2.68)	<0.001
Region (urban)				
Rural	0.67 (0.61,0.72)	<0.001	0.85 (0.74,0.97)	0.02
Family size (<4)				
>4	0.78 (0.73,0.84)	<0.001	0.90 (0.82,0.98)	0.04
Sweetened beverages (non-daily)				
daily	0.70 (0.62, 0.80)	<0.001	0.82(0.70,0.95)	0.01
Living with parent (none of them)				
One of them	1.30 (0.91,1.86)	0.14	1.50 (0.93,2.42)	0.09
Both of them	1.43 (1.05,1.97)	0.02	1.36 (0.92,2.02)	0.11
Smoking status (no smoker)				
Only passive smoker	0.74 (0.68,0.80)	< 0.001	0.76 (0.70,0.83)	< 0.001
Only active smoker	0.39 (0.25, 0.61)	< 0.001	0.45 (0.27, 0.73)	0.001
Passive and active smoker	0.53 (0.41,0.70)	<0.001	0.60(0.45,0.79)	<0.001
Age (year)	1.04 (1.02, 1.05)	<0.001	1.03 (1.02,1.05)^a^	<0.001

^a^Statistically significant

*OR* odds ratio, *CI* confidence interval, *Q* quantile, *FH* family history

To evaluate the relationship between SES and oral health for identifying particular factors associated with SES that contribute to the risk of dental caries, analysis of the socio-economic factors which cause the gap in tooth brushing between the first and fifth quintiles showed that only 3 % of the difference was explained by the factors considered in the study, and 17 % remained unknown. Residence area, family size, and smoking status made a significant contribution to the gap between the first and last SES groups. Residence area ( −2.01 (95 % CI: −3.46, −0.55)) was along with the maximum levels of gaps between SES categories.

Associations of independent variables with tooth brushing in logistic regression analysis are provided in Table 3.

**Table 3:** Decomposition of the gap in teeth brushing between the first and fifth quintiles of socio-economic status

	Percent (95 % CI)	*p*-value
Prevalence in the fifth quintile	58.20 (56.21,60.19)*	< 0.001
Prevalence in the first quintile	78.66 (77.03,80.29)*	< 0.001
Differences (total gap)	−23.03 (−22.96,−17,89) *	< 0.001
Due to endowments (explained)		
Age	−0.12 (−0.28,0.04)	0.14
Sex	0.35 (−0.14,0.83)	0.16
Region	−2.01 (−3.46,−0.55)*	0.01
Family size	−1.73 (−2.97,−0.50)*	0.01
Birth order	0.20 (−1.13,1.53)	0.77
Sweetened beverages	−0.09 (−0.21,0.03)	0.13
Living with parents	−0.06 (−0.20,0.09)	0.43
Smoking status	−0.37 (−0.68,−0.06) *	0.02
Subtotal gap	−3.83 (−5.94,−1.73)*	< 0.001
Due to coefficients (unexplained)		
Age	−6.15 (−15.04,2.74)	0.18
Sex	8.12 (0.65,15.59) *	0.03
Region	−12.78 (−21.71,−3.86)*	0.01
Family size	−2.60 (−11.96,6.77)	0.59
Birth order	2.76 (−0.79,6.30)	0.13
Sweet meat	0.72 (−0.05,1.50)	0.07
Living with parent	0.17 (−0.27,0.62)	0.45
Smoking status	1.15 (−1.04,3.34)	0.30
Constant	−8.02 (−25.30,2.25)	0.36
Subtotal gap	−16.63(−19.84,−13.42)*	< 0.001

*p-value 0.05

## Discussion

This study demonstrated considerable differences in oral health-related behaviors between high and low SES groups of Iranian children and adolescents; thus, the prevalence of tooth brushing increased with improvement of SES.

There was 20 % difference in prevalence of tooth brushing between the first and fifth quintiles. In addition, individuals in quintile 5 families had significantly higher odds of tooth brushing compared with those in quintile 1 families.

The association between birth order, family size, and living area with tooth brushing showed that children and adolescents with higher order of birth and larger family size brushed less frequently, 24 % and 22 %, respectively, as well as those living in rural areas (33 %). In contrast, tooth brushing was 2.31 times more prevalent in girls; with increasing the age of population, the odds of tooth brushing increased. Moreover, analysis of the socio-economic factors, which causes the gap in tooth brushing between the first and fifth quintiles, showed that only 3 % of the difference was explained by the factors considered in the study; however, 17 % remained unknown.

Most studies confirm the association between socio-economic status and dental caries in children and adolescents [7–11, 30–32]. In a study conducted in Scotland, the inequalities in tooth brushing were examined among adolescents, revealing that socio-economic inequalities in tooth brushing were significant for both boys and girls at all ages [12]. Mashoto provided a survey on socio-demographic disparity in oral health among adolescents in Tanzania; adolescents in the poorest wealth category presented poor oral hygiene behavior who were more frequently no users of tooth brushing compared with the least poor wealth category [13]. In another study in Belgium, oral health-related lifestyle behaviors were investigated among children and adolescents, showing that children from lower-SES families had less frequent tooth-brushing [33]. Some studies in the United Kingdom had similar findings. It was found that children in lower socio-economic families were more likely to have late tooth brushing and brush less frequently [34–36].

On the other hand, oral health behaviors of children in low and high socioeconomic status families were evaluated for a period of 9 years in a study conducted in Iowa. In contrast, it was shown that there were virtually no differences at any time point between the two groups regarding tooth-brushing frequency [37].

Third National Oral Health Survey among 5-year-olds in four Chinese provinces revealed a significant gradient in children’s Decayed, Missing, and Filled Teeth (DMFT) by household income which increased from 2.63 in the highest income group to 4.70 in the lowest income group. It was considerable that parental education was not significantly related to childhood dental caries [38]. Another study on adolescent sample of Pennsylvania showed that lower SES was associated with higher prevalence of DMFT and severe caries. Lower SES was associated with lower rates of brushing, less use of sealants, and receiving less recent dental services [39]. Using seven comparable cross-sectional data of nationally representative samples of 11- to 15-year-olds in Denmark, the absolute social inequality increased from 7.7 % in 1991 to 14.6 % in 2014 as the prevalence difference between low and high social class. The relative social inequality assessed by odds ratios for infrequent tooth brushing also increased from 1991 to 2014 [40]. Regarding the related factors, an investigation on 11- to 15-year-olds in Denmark revealed that, comparing with girls, boys in lower social class had higher odds ratio of infrequent tooth brushing than girls: 1.98 (95 % confidence interval 1.62–2.41) vs. 1.80 (1.53–2.24). Also, immigrants and descendants had higher odds compared to adolescents of Danish origin. Analyses of the combined effect of social class and migration status showed that the social gradient in tooth brushing habits among ethnic Danes was not found among groups of immigrants and descendants [41].

Considering other studies, analysis of oral health behaviors changes over time in Brazilians revealed that the prevalence of oral health behaviors followed an increasing trend; however, these changes were not related to maternal education inequalities [42].

The strengths of the study are as follows. First, the current study is one of the first few of its kind in evaluating the socio-economic inequality in oral health behavior among Iranian children and adolescents. Another advantage is the large nationwide study population which increased the chance of finding specific and statistically significant differences. Furthermore, the other strength of the study is its novelty in selection of pediatric and adolescence age group. Finally, the association between tooth brushing and socio-economic status of the study population was considered using the Oaxaca-Blinder decomposition method and well-conceptualized measures of socio-economic inequality in health. The use of measures of absolute inequality including slope index of inequality (SII), relative inequality such as concentration index (C), and regression-based rate ratios between the groups well-suited the objective of the study.

The findings should be considered in the context of potential limitations. The major limitation was cross-sectional nature of the study; thus, a causal relationship cannot be inferred from the current findings, and longitudinal studies are required to examine the causality and clinical importance of the outcomes. In addition, the teeth could not be examined and data could not be collected regarding oral hygiene status; however, tooth brushing was used as a single marker for evaluation of oral health. Considering the factors of inequality, there was a low portion of explained factors, i.e. other socio-economic factors might have affected oral health behavior.

Monitoring socio-economic inequality in health, including oral health, is considered important in formulating appropriate public policies. Population oral health policies aiming to improve the overall oral health of the population should target socio-economic inequality. Furthermore, those policies may need to be modified to suit different socio-economic groups.

## Conclusion

Overall, the current analysis revealed socio-economic inequality in oral health behavior of Iranian children and adolescents. Since oral hygiene is essential to oral health, it is wise to provide the population with adequate education and training on children’s oral health behavior and its relationship with dental caries. It is helpful to address factors that influence oral health in order to develop and implement complementary public health actions. Prevention programs and policies for primitive and primary prevention of oral diseases should aim to increase the oral health awareness and improve oral health.

## Appendix

**Table 4:** List of questions regarding oral health behavior according to Global School-Based Student Health Survey (GSHS) questionnaires

Question	Response
Oral health behaviors	
How often do you brush your teeth?	1. More than once a day
	2. Once a day
	3. At least once a day
	4. Once a week
	5. Less than once a week
	6. Never
Demographic variables	
Parental education	1. Illiterate
	2. Elementary
	3. Secondary
	4. Diploma
	5. Bachelor
	6. Master or higher
Type of home	1. Private
	2. Rented
Maternal job	1. Unemployed
	2. Worker
	3. Employee
	4. Farmer
	5. Self-employment
Paternal job	1. Unemployed
	2. Worker
	3. Employee
	4. Farmer
	5. Self-employment
Socioeconomic status	Highest
	Second highest
	Moderate
	Second lowest
	Lowest

## Abbreviations

CASPIAN: Childhood and Adolescence Surveillance and Prevention of Adult Non-communicable Disease
CI: Confidence interval
OR: Odds ratio
PCA: Principle component analyses
SES: Socioeconomic status
SII: Slope index of inequality
WHO: World Health Organization

## References

[1] Fiscella K, Williams DR (2004). Health disparities based on socioeconomic inequities: implications for urban health care. Acad Med.

[2] Drewnowski A, Specter S (2004). Poverty and obesity: the role of energy density and energy costs. Am J Clin Nutr.

[3] Adler NE, Boyce T, Chesney MA, Cohen S, Folkman S, Kahn RL, Syme SL (1994). Socioeconomic status and health: the challenge of the gradient. Am Psychol.

[4] Health UDo, Services H: Oral health in America: a report of the Surgeon General. May 2000. Complete report available at: http://www.surgeongeneral.gov/library/reports/oralhealth 2008.

[5] Pihlstrom BL, Tabak L (2005). The National Institute of Dental and Craniofacial Research: research for the practicing dentist. J Am Dent Assoc.

[6] Do LG, Scott JA, Thomson WM, Stamm JW, Rugg-Gunn AJ, Levy SM, Wong C, Devenish G, Ha DH, Spencer AJ (2014). Common risk factor approach to address socioeconomic inequality in the oral health of preschool children–a prospective cohort study. BMC Public Health.

[7] Reisine ST, Psoter W (2001). Socioeconomic status and selected behavioral determinants as risk factors for dental caries. J Dent Educ.

[8] Gillcrist JA, Brumley DE, Blackford JU (2001). Community socioeconomic status and children’s dental health. J Am Dent Assoc.

[9] Källestål C, Wall S (2002). Socio‐economic effect on caries. Incidence data among Swedish 12–14‐year‐olds. Community Dent Oral Epidemiol.

[10] Watt R, Sheiham A (1999). Inequalities in oral health: a review of the evidence and recommendations for action. Br Dent J.

[11] Do L, Spencer A, Slade G, Ha D, Roberts-Thomson K, Liu P (2010). Trend of income-related inequality of child oral health in Australia. J Dent Res.

[12] Levin K, Currie C (2009). Inequalities in toothbrushing among adolescents in Scotland 1998–2006. Health Educ Res.

[13] Mashoto KO, Astrom AN, Skeie MS, Masalu JR (2010). Socio-demographic disparity in oral health among the poor: a cross sectional study of early adolescents in Kilwa district, Tanzania. BMC Oral Health.

[14] Sadinejad M, Kelishadi R, Qorbani M, Shahsanai A, Motlagh ME, Ardalan G, Heshmat R, Keikha M (2014). A nationwide survey on some hygienic behaviors of Iranian children and adolescents: the CASPIAN-IV study. Int J Prev Med.

[15] Kelishadi R, Heshmat R, Motlagh M, Majdzadeh R, Keramatian K, Qorbani M, Taslimi M, Aminaee T, Ardalan G, Poursafa P (2012). Methodology and early findings of the third survey of CASPIAN study: a National School-based surveillance of students’ high risk behaviors. Int J Prev Med.

[16] Kelishadi R, Mirmoghtadaee P, Qorbani M, Motlagh ME, Heshmat R, Taslimi M, Mahmoudarabi M, Ardalan G, Larijani B (2013). Tooth brushing and cardiometabolic risk factors in adolescents: is there an association? The CASPIAN-III study. Int J Prev Med.

[17] Jann B. The Blinder-Oaxaca decomposition for linear regression models. The Stata Journal. 2008;8(4):453–79.

[18] Borooah VK, Iyer S (2005). The decomposition of inter-group differences in a logit model: Extending the Oaxaca-Blinder approach with an application to school enrolment in India. J Econ Soc Meas.

[19] Fournier M (2005). Exploiting information from path dependency in Oaxaca–Blinder decomposition procedures. Appl Econ Lett.

[20] Jiménez-Rubio D, Hernández-Quevedo C (2011). Inequalities in the use of health services between immigrants and the native population in Spain: what is driving the differences?. Eur J Health Econ.

[21] O’Donnell OA, Wagstaff A. Analyzing health equity using household survey data: a guide to techniques and their implementation. Washington DC: World Bank Publications; 2008.

[22] Abdi H, Williams LJ (2010). Principal component analysis. Wiley Interdiscip Rev: Comput Stat.

[23] Jolliffe I. Principal component analysis: Wiley Online Library; 2002.

[24] Emamian M, Zeraati H, Majdzadeh R, Shariati M, Hashemi H, Jafarzadehpur E, Fotouhi A (2013). Economic inequality in presenting near vision acuity in a middle-aged population: a Blinder-Oaxaca decomposition. Br J Ophthalmol.

[25] Emamian M, Zeraati H, Majdzadeh R, Shariati M, Hashemi H, Fotouhi A (2011). The gap of visual impairment between economic groups in Shahroud, Iran: a Blinder-Oaxaca decomposition. Am J Epidemiol.

[26] Koolman X, van Doorslaer E (2003). On the interpretation of a concentration index of inequality. Health Econ.

[27] SHA H, Debbie A, Shaw GD. The Handbook of Inequality and Socioeconomic Position: Concepts and measures. J Soc Policy. 2009;38:545.

[28] Pamuk E (1985). Social class inequality in mortality from 1921 to 1972 in England and Wales. Popul Stud.

[29] Jann B (2008). A Stata implementation of the Blinder-Oaxaca decomposition. Stata J.

[30] Locker D (2000). Deprivation and oral health: a review. Community Dent Oral Epidemiol.

[31] Petersen PE (2005). Sociobehavioural risk factors in dental caries–international perspectives. Community Dent Oral Epidemiol.

[32] Slade G, Sanders A, Bill C, Do L (2006). Risk factors for dental caries in the five-year-old South Australian population. Aust Dent J.

[33] Vereecken CA, Maes L, De Bacquer D (2004). The influence of parental occupation and the pupils’ educational level on lifestyle behaviors among adolescents in Belgium. J Adolesc Health.

[34] Maes L, Vereecken C, Vanobbergen J, Honkala S (2006). Tooth brushing and social characteristics of families in 32 countries. Int Dent J.

[35] Gregory J, Lowe S, Bates CJ, Prentice A, Jackson L, Smithers G, Wenlock R, Farron M. National Diet and Nutrition Survey: young people aged 4 to 18 years; volume 1: report of the diet and nutrition survey. Stationery Office; 2000.

[36] Silver D (1992). A comparison of 3-year-olds’ caries experience in 1973, 1981 and 1989 in a Hertfordshire town, related to family behaviour and social class. Br Dent J.

[37] Hamasha AA-H, Warren JJ, Levy SM, Broffitt B, Kanellis MJ (2006). Oral health behaviors of children in low and high socioeconomic status families. Pediatr Dent.

[38] Guan Y, Zeng X, Tai B, Cheng M, Huang R, Bernabé E (2015). Socioeconomic inequalities in dental caries among 5-year-olds in four Chinese provinces. Community Dent Health.

[39] Polk DE, Weyant RJ, Manz MC (2010). Socioeconomic factors in adolescents’ oral health: are they mediated by oral hygiene behaviors or preventive interventions?. Community Dent Oral Epidemiol.

[40] Holstein B, Bast L, Brixval C, Damsgaard M (2015). Trends in social inequality in tooth brushing among adolescents: 1991-2014. Caries Res.

[41] Bast L, Nordahl H, Christensen L, Holstein B (2015). Tooth brushing among 11-to 15-year-olds in Denmark: combined effect of social class and migration status. Community Dent Health.

[42] Freire MCM, Jordão LMR, Malta DC, de Araújo Andrade SSC, Peres MA (2015). Socioeconomic inequalities and changes in oral health behaviors among Brazilian adolescents from 2009 to 2012. Rev Saude Publica.

